# Quality assurance and best research practices for non-regulated veterinary clinical studies

**DOI:** 10.1186/s12917-017-1153-x

**Published:** 2017-08-16

**Authors:** R. Davies, C. London, B. Lascelles, M. Conzemius

**Affiliations:** 1 College of Veterinary Medicine, University of Minnesota, St. Paul, MN USA; 2 Cummings School of Veterinary Medicine, Tufts University, North Grafton, MA USA; 3 Comparative Pain Research Laboratory, Department of Clinical Sciences, College of Veterinary Medicine, North Carolina State University, Raleigh, NC USA; 4 Comparative Medicine Institute, North Carolina State University, Raleigh, NC USA

## Abstract

Veterinary clinical trials generate data that advance the transfer of knowledge from clinical research to clinical practice in human and veterinary settings. The translational success of non-regulated and regulated veterinary clinical studies is dependent upon the reliability and reproducibility of the data generated. Clinician-scientists that conduct veterinary clinical studies would benefit from a commitment to research quality assurance and best practices throughout all non-regulated and regulated research environments. Good Clinical Practice (GCP) guidance documents from the FDA provides principles and procedures designed to safeguard data integrity, reliability and reproducibility. While these documents maybe excessive for clinical studies not intended for regulatory oversight it is important to remember that research builds on research. Thus, the quality and accuracy of all data and inference generated throughout the research enterprise remains vulnerable to the impact of potentially unreliable data generated by the lowest performing contributors. The purpose of this first of a series of statement papers is to outline and reference specific quality control and quality assurance procedures that should, at least in part, be incorporated into all veterinary clinical studies.

## Introduction

Veterinary clinical studies are designed to determine whether a medical intervention (e.g. device, treatment, approach) is safe and effective when used in client-owned animals. While such studies are typically performed in veterinary patients with spontaneous disease (as opposed to experimentally induced models), occasionally studies are performed in healthy client owned dogs (e.g. for disease prevention). Recently, in addition to benefiting animal health, an increasing number of clinical trials in veterinary patients are being undertaken to evaluate a novel therapeutic or device prior to initiation of human clinical trials (known as comparative and/or translational trials). Such studies are based on the premise that spontaneous disease in veterinary patients more closely recapitulates similar human diseases and therefore, data generated from these trials would be more informative than that generated using induced models of disease. Ultimately, the overarching goal of these efforts is to generate data that advance the transfer of knowledge from clinical research to clinical practice in human and veterinary settings [1]. Veterinary clinical trials are also an important component of the ‘one health’ research continuum where complex health interactions among animals, humans and the environment are recognized as key targets for affecting and advancing global health [2].

Non-regulated and regulated veterinary clinical studies lead to medical advances that improve the health of animals, people and the environment; however, the success of translational and comparative medicine is dependent on the ability of scientists to ensure that the data generated are reliable and reproducible. Research builds upon research; therefore, when expectations related to data quality and study conduct are not standardized, the quality and accuracy of all data and inference generated throughout the research enterprise remains vulnerable to the impact of potentially unreliable data generated by the lowest performing contributors.

To mitigate this risk, clinician-scientists that conduct non-regulated and regulated veterinary clinical studies would benefit from a commitment to research quality assurance and best practices throughout all non-regulated and regulated research environments.

## Conducting regulated veterinary clinical trials

The Food and Drug Administration (FDA) provides internationally harmonized guidance on the ‘design and conduct of all clinical studies of veterinary products in the target species’. (FDA Guidance for Industry: Good Clinical Practice, (GCP; VICH GL9. No 85) [3]. This guidance document is intended to ensure that veterinary clinical studies incorporate the recommended best practices that support the integrity of the study data, and protection of people, animals, the food supply and the environment.

The GCP guidelines established for use with veterinary clinical studies is not the same as the GCP guidelines for clinical trials performed in humans [4, 5]. Nevertheless, both standards include quality assurance (QA) principles and procedures designed to safeguard data integrity, reliability and reproducibility. Adherence to the respective GCP standards is mandated for research conducted in animals or humans when the clinical data are intended to be submitted to regulatory authorities (FDA) for approval. These GCP guidelines provide clear expectations, a consistent approach for conducting ethical, responsible and reliable scientific research, and an established route to compliance. Adherence to them reinforces appropriate research behaviors, supports research quality, and facilitates the development and sustainability of research environments where research and data management best practices are routine. There are other federal (and state) regulations and guidelines that may also be applicable to veterinary, human and translational science including Good Manufacturing Practice (GMP), Good Laboratory Practices (GLP) and others within the Code of Federal Regulation (21 CFR) [6].

## Conducting non-regulated veterinary clinical trials

Many foundational (basic and discovery), translational, and one-health research investigations are conducted in non-regulated research environments because the studies are exploratory and data are not intended to be submitted to regulatory authorities. As a result, foundational research is conducted in an ad hoc manner that is highly dependent on individual investigators and is rarely subject to external monitoring prior to entering the publication review process. This inconsistent approach is problematic because research in human and veterinary medicine is not unidirectional. All data along the research spectrum (non-regulated through regulated, discovery through translational) have the potential to advance, divert, or limit scientific progress at all stages. If the quality of the data is questionable in the foundational studies and if these data remain unchallenged and uncorrected, progress at all stages will be impeded - sometimes for years to come.

## Research quality

All research data streams (and their associated research inference and outcomes) are subject to the traditional gatekeepers of scientific quality such as research mentoring, peer review, and the self-correcting nature of science. However, the scientific community and the public it serves, are increasingly being presented with troubling indications that these gatekeepers are failing to ensure that research data are reliable, reproducible and lead to improvements in health outcomes [7–12]. These and other reports illustrate the frequency and high cost of irreproducible research, questionable research practices and research waste [13–17]. As a result, funding, publishing and quality assurance organizations are exploring ways to ensure the quality of the data they fund, publish and support. The National Institute of Health (NIH) has expanded guidelines to enhance rigor and reproducibility (https://www.nih.gov/research-training/rigor-reproducibility) in the scientific research they fund [15, 18]. Scientific journals have established specific policies designed to encourage the submission of research reports that are reproducible, robust, and transparent [19] and the American Society for Quality (ASQ) has recommended the establishment of a national quality standard for biomedical research in drug development [20].

Funding, publishing and QA associations all have an obvious incentive to engage in efforts to improve scientific research outcomes. However, individual scientists are directly responsible for the generation, quality, integrity and security of experimental data, in addition to the on-going mentoring of research trainees. Their perspective and participation is required to develop effective strategies that will advance translational medicine and improve research outcomes [14]. Individual scientists must be accountable for the quality of their work and the integrity of their data by providing credible assurances that data are robust, reliable and transparent so that their contributions effectively support the entire research enterprise.

Scientists conducting veterinary clinical trials frequently work in non-regulated research settings where flexibility, innovation and creativity are highly valued because these characteristics facilitate learning, self-correction, redirection, and serendipity. In comparison, veterinary clinical trials conducted within regulated research programs are partially constrained to meet regulatory requirements established to ensure patient safety and maintain data integrity. In spite of these differences, the scientist-driven development of a common approach to basic data quality that spans the non-regulated and regulated veterinary clinical trial spectrum would be an effective strategy for demonstrating data quality and enhancing research reliability.

## Maintaining research quality in non-regulated research

Data quality across the non-regulated to regulated research spectrum could be maintained through the establishment of a national quality standard with requirements that should be (voluntary) or must be (regulatory) met. The American Society for Quality (ASQ) advocates for the establishment, implementation, and maintenance of a quality management system for biomedical research in drug development that is based on international quality assurance (QA) standards [20]. Others also recommend adapting existing International Organization for Standardization (ISO) standards for research laboratories [21]. While this approach sets a justifiably high bar for research conduct, it may be difficult for individual research scientists to meet the requirements while working within typical non-regulated research environments where QA support and expertise are historically rare.

Consistent data quality could also occur through the implementation of an individualized, voluntary and sustainable approach that does not dramatically expand the scope of work, constrain creativity and flexibility, or commit to a ‘one-size fits all’ system of quality management. Strategies for integrating best practices into basic research environments are not new, [20–24] and selected resources designed to help scientists improve research data quality and integrity are listed in Table 1 In 2006, the World Health Organization (WHO) updated their freely available ‘Handbook: Quality Practices in Basic Biomedical Research (QPBR)’ to encourage the adoption of QA principles in basic research [25]. More recently, the Research Quality Association (RQA) has produced ‘Quality in Research, Guidelines for working in non-regulated research’ which is available for a nominal fee [26]. In addition, ‘Good Research Practices’, (GRP) [22, 27], and the use of electronic notebooks [23] propose reasonable strategies for integrating QA within the non-regulated drug discovery process. A basic research QA toolkit that provides tools and templates for integrating QA best practices into research settings is also freely available [27].

**Table 1:** Resources for integrating Quality Assurance Best Practices into non-regulated research

Reference	Title	Stated Purpose
[25]	TDR Handbook: Quality Practices in Basic Biomedical Research, (QPBR)	‘Provide institutions and researchers with the necessary tools for the implementation and monitoring of quality practices in their research, thus promoting the credibility and acceptability of their work. The handbook highlights non-regulatory practices that can be easily institutionalized with very little extra expense’.
[26]	RQA: Quality in Research Guidelines for working in non-regulated research	‘to facilitate the stepwise and straightforward development of a value-adding Quality System into any research institute.
[27]	Michelson Prize & Grants Research Quality Assurance Toolkit	A basic QA toolkit designed to facilitate best practices in research and data management. Tools and templates that facilitate the development of effective records (personnel, equipment, methods, supplies/reagents, and data) throughout the data life cycle are provided.
[29]	RQA Quality Systems Workbook [34]	‘to provide tools and a practical approach to develop a Quality System that works for the user’
[20]	ASQ TRI-2012: Best Quality Practices for Biomedical Research in Drug Development	‘This technical report identifies important quality management system elements for non-regulated biomedical research in drug development in order to ensure credibility of biomedical research results.’
[21]	Quality assurance mechanisms for the unregulated research environment.	
[22]	Quality: an old solution to new discovery dilemmas.	Implementation of Good Research Practices for the early phase, non-regulated drug discovery research environment.
[35]	A novel audit model for assessing quality in non-regulated research	

Unfortunately, these sound principles and practices have not been adopted in most academic environments because research trainees (and the faculty that mentor them) are not specifically educated in QA principles and procedures. QA support is rare within most non-regulated research settings and funding agencies do not typically require adherence to QA best practices. In spite of these challenges, scientists should consider implementing research QA as a timely and reasonable strategy for demonstrating the quality of their data and the credibility of their research. In addition, there may be a competitive funding advantage for those that adopt and demonstrate research QA best practices.

## Resources for improving and demonstrating research quality

The resources listed in Table 1 were developed to maintain research flexibility and support (and monitor) data quality and reconstruction. They encourage basic research scientists to integrate QA activities and commit to good documentation practices within their research environment in order to ensure the consistent management (policies, processes and procedures) of personnel, equipment, methods (procedures), reagents, supplies, and data. Some resources (References [25, 27]) include downloadable and adaptable documentation tools and templates for use within specific research settings.

An individual, a team (specific research project based), a program, a college or a private hospital could use the resources identified in Table 1 to design a quality management system or integrate specific quality assurance best practices. Research mentors that wish to establish a full research Quality Management System (QMS) could do so by using these resources to design a sustainable integration and implementation approach. Alternatively, clinician-scientists could begin by selecting specific best practices that would directly mitigate potential risks within their research projects. In a multi-institutional clinical study, for instance, investigators may choose to create standard operating procedures (SOPs) for routine tasks to ensure that all scientists in all areas are consistently performing critical procedures. In the case of a routine procedure such as blood sampling, the SOP might include documentation of patient data, when the blood was collected, who collected the blood, and uniform storage and mailing procedures to a central laboratory. Others may choose to establish a consistent approach to laboratory notebooks and data management so that data are secure, archived, retrievable and do not change over time. Investigators that depend upon electronic data capture may choose to focus on data security, verification and accurate transfer across users and networks. Equipment management is critical for the generation of reliable data in all research settings. Therefore, a reasonable first step for improving data reliability would be to develop consistent procedures for maintaining and managing critical research equipment. Uncertain reagent quality and characterization is a known confounder contributing to research irreproducibility [28]. Therefore, scientists using critical reagents should implement good documentation practices for the receipt, characterization and use of research reagents and supplies.

Using this voluntary and risk-based approach, a research QA implementation plan will be scientist-driven and sustainable over time. Investigators that describe the research QA measures they have adopted will be providing credible evidence within their manuscripts, grant applications, and study reports that their data have been generated and maintained under conditions that support data rigor and reliability. In addition, the integration of these practices will create a laboratory culture where research QA is routine, providing important opportunities for expanding QA expertise among current and future research scientists.

## Establishing and maintaining research QA best practices

If possible, clinical investigators should consider integrating research QMS using the resources listed in Table 1. In addition to these resources, clinical investigators should explore opportunities to seek help and advice from QA professionals who are supporting regulated research within their research institutions.

Alternatively, if the scope of a full QMS is unrealistic, scientists should commit to QA of best practices that reduce specific risks to data integrity and support the data and records collected within their non-regulated veterinary clinical trials. Table 2 provides a research documentation checklist to help scientists determine whether they have the records needed to: (1) support data integrity throughout the research data life cycle; (2) readily reconstruct research data; and (3) provide credible evidence (documentation) demonstrating the quality of their research management. Some resources listed in Table 1 (References [25, 27]) contain tools and templates useful for creating the records recommended in Table 2.

**Table 2:** Research Documentation Checklist

Project Management: Ensure that research objectives, approach, timeline and budget are planned, communicated and understood.	Yes	No
1. Project plan (roles and responsibilities, objectives, timeline)		
2. Research review plan		
3. Research publication plan		
Personnel Records: Ensure that research records can be traced to competent and appropriate personnel	Yes	No
1. Job descriptions, resumes or CVs		
2. Signature & initials identification log		
3. Training and ongoing competency (procedures, policies, methods, equipment) records		
Critical Equipment Records: Ensure that research records can be traced to well managed and fully operational equipment	Yes	No
1. Equipment inventory log (unique identification)		
2. Equipment use, maintenance, verification and calibration records		
3. Standard Operating Procedures (SOPs) for use, care and management of equipment		
4. Computer systems used to capture, process, generate and report data should be secure, working as expected and fit for their intended purpose.		
Method/Procedure Records: Ensure that research data can be traced to methods or procedures that are well described, working as expected, and fit for their intended purpose	Yes	No
1. SOPs for routine research methods		
2. Method validation records		
3. On-going quality control records		
Standard Operating Procedures: Ensure that procedures are performed consistently, revised as needed and maintained as historical records	Yes	No
1. Routine procedures: research methods, equipment use, personnel training, data and research management (lab notebooks, research review, reagents and supplies, data (paper and electronic) collection, use and security)		
2. Document management (creation, revision, archiving)		
3. SOP linkages to associated recording forms		
Research Records (paper/electronic): Ensure that research data and work (who, what, where, when, how) can be fully reconstructed	Yes	No
1. Reagent inventory, reagent characterization, verification and preparation records (receipt, verification, storage, expiration and disposition), supply records		
2. Facilities data (temperature, water/air quality, emergency preparedness) if quality critical		
3. Unique identification records for research subjects and samples.		
4. Sample handling and storage procedures		
5. Re-constructable records (accurate, legible, contemporaneous, original, attributable, unchanging, readily retrievable, secure)		
6. Error management procedures (detecting, recording, managing errors, outliers and non-conforming data)		

Once scientists have successfully integrated a QMS or a targeted approach to a quality commitment, they must monitor their program to ensure compliance and to capture opportunities for continuous improvement within their research management program. Approaches to monitoring quality activities may include self- team- or peer-assessment, the establishment of QA metrics, or the integration of other types of research audit exercises. Finally, scientists should communicate their approach to research quality within their grant proposals and research reports so that other scientists can evaluate the rigor of their research and data management program.

## Study design

It is important to note that institution of QA practices will likely positively influence your study designs. For all clinical studies, an outline, or checklist, that addresses key methodological components to consider may be utilized to ensure that the trial possesses fundamental components necessary to be completed [29, 30]. This will improve the quality of your research manuscript and may be required as a reporting guideline by a scientific journal for publication [31]. While a basic guide is presented in Table 3, it is important to note that this can be expanded to include other topics investigators may want to routinely consider when performing a study. For example, most would consider animal care review, randomization and blinding procedures when building a clinical study, but often the details of the subsequent statistical review are not included. It is ideal to document a specific statistician, review the research procedures with the statistician and document what statistical methods might be used prior to the initiation of a trial. One could also consider expanding the statistical component of the outline by incorporating topics to potentially be incorporated in the data evaluation such as *p*-value vs. effect size, statistical vs. clinical significance and defining treatment success and failure [32]. This facilitates the documentation of Number Needed to Treat, Number Needed to Harm, Absolute Risk Reduction and Absolute Risk Increase of an intervention [33]. While utilizing a study design outline increases time invested in a study prior to initiation, it decreases the likelihood of overlooking critical methods of unanticipated bias, improves communication of methods to all study personnel and shortens the data evaluation and manuscript writing processes.

**Table 3:** Basic outline that can be used to build a clinical study

1. Study Personnel	
2. Background	
3. Objectives	
4. Study Design	
a. Study Type	
b. Study Overview	
c. Treatment Groups	
d. Randomization Procedures	
e. Blinding Procedures	
5. Intervention and Placebo Details	
6. Population Studied	
a. Institutional Protocol Review	
b. Informed Owner Consent	
c. Animal Identification	
d. Inclusion Criteria	
e. Exclusion Criteria	
f. Removal and Rescue Criteria	
7. Assessments	
a. Veterinary Outcome Measures	
b. Owner Outcome Measures	
c. Patient Biologic Measures	
8. Adverse Events	
9. Statistical Analysis	
10. Data Collection, Security and Independent Review	
11. Protocol Deviations and Changes	

## Conclusions

Translational or comparative science on behalf of animal, human and environmental health is not linear or unidirectional. Outcomes along the entire research spectrum require constant scrutiny as new data inform ongoing work in regulated and non-regulated research efforts. This mutual dependency means that scientists *must commit to a shared vision of research rigor and research conduct* to minimize the risk of inconsistent data quality and irreproducible research outcomes. A voluntary commitment to research best practices designed to support data integrity and reliability provides scientists with a roadmap for conducting quality research, and the opportunity to support the work of their peers by ensuring a consistent and reliable data stream throughout the research continuum. Clinician-scientists have the most to gain by voluntarily establishing the characteristics of this data stream so that they can define sustainable approaches, demonstrate the quality of their research and warrant continued funding for the important work they do.
